# Unveiling the Antiviral Properties of Panduratin A through SARS-CoV-2 Infection Modeling in Cardiomyocytes

**DOI:** 10.3390/ijms25031427

**Published:** 2024-01-24

**Authors:** Aung Khine Linn, Suwimon Manopwisedjaroen, Phongthon Kanjanasirirat, Suparerk Borwornpinyo, Suradej Hongeng, Phetcharat Phanthong, Arunee Thitithanyanont

**Affiliations:** 1 Excellent Center for Drug Discovery, Faculty of Science, Mahidol University, Bangkok 10400, Thailand; agkhinelinn@protonmail.com (A.K.L.); bsuparerk@gmail.com (S.B.); 2 Department of Microbiology, Faculty of Science, Mahidol University, Bangkok 10400, Thailand; swiboonut@gmail.com; 3 Department of Pathobiology, Faculty of Science, Mahidol University, Bangkok 10400, Thailand; phongthon.kan@mahidol.edu; 4 Department of Biotechnology, Faculty of Science, Mahidol University, Bangkok 10400, Thailand; 5 Department of Pediatrics, Faculty of Medicine, Ramathibodi Hospital, Mahidol University, Bangkok 10400, Thailand; suradej.hon@mahidol.ac.th; 6 Department of Anatomy, Faculty of Science, Mahidol University, Bangkok 10400, Thailand

**Keywords:** SARS-CoV-2, cardiomyocytes, drug screening, antiviral agent, panduratin A

## Abstract

Establishing a drug-screening platform is critical for the discovery of potential antiviral agents against SARS-CoV-2. In this study, we developed a platform based on human induced pluripotent stem cell-derived cardiomyocytes (iPSC-CMs) to investigate SARS-CoV-2 infectivity, with the aim of evaluating potential antiviral agents for anti-SARS-CoV-2 activity and cardiotoxicity. Cultured myocytes of iPSC-CMs and immortalized human cardiomyocyte cell line (AC-16) were primarily characterized for the expression of cardiac markers and host receptors of SARS-CoV-2. An infectivity model for the wild-type SARS-CoV-2 strain was then established. Infection modeling involved inoculating cells with SARS-CoV-2 at varying multiplicities of infection (MOIs) and then quantifying infection using immunofluorescence and plaque assays. Only iPSC-CMs, not AC16 cells, expressed angiotensin-converting enzyme 2 (ACE-2), and quantitative assays confirmed the dose-dependent infection of iPSC-CMs by SARS-CoV-2, unlike the uninfectable AC16 cells lacking the expression of ACE2. Cytotoxicity was evaluated using MTT assays across a concentration range. An assessment of the plant-derived compound panduratin A (panA) showed cytotoxicity at higher doses (50% cytotoxic concentration (CC_50_) 10.09 μM) but promising antiviral activity against SARS-CoV-2 (50% inhibition concentration (IC_50_) 0.8–1.6 μM), suppressing infection at concentrations 10 times lower than its CC_50_. Plaque assays also showed decreased viral production following panA treatment. Overall, by modeling cardiac-specific infectivity, this iPSC-cardiomyocyte platform enables the reliable quantitative screening of compound cytotoxicity alongside antiviral efficacy. By combining disease pathogenesis and pharmacology, this system can facilitate the evaluation of potential novel therapeutics, such as panA, for drug discovery applications.

## 1. Introduction

Induced pluripotent stem cell (iPSC)-derived cardiomyocytes are powerful tools for drug screening and discovery [1]. With the ability to offer a scalable and cost-effective way to study human cardiac biology, these cells have been used to study cardiac diseases and drug toxicity and to develop new treatments. Additionally, they have been employed in high-throughput screening assays to identify new drugs and assess their efficacy and safety [2,3]. In the search for potential antiviral agents, drug discovery screens are powerful tools for rapidly identifying active compounds. These screens provide a systematic approach to testing the effects of thousands of compounds in a single experiment and can be used to identify active compounds that can interfere with a virus’s replication process [4]. This is a critical step in the drug discovery process because the identified compounds can be further investigated to determine their potential efficacy against viruses. 

The novel coronavirus SARS-CoV-2 caused a pandemic of unprecedented scale in 2020 [5]. Despite the reductions in viral infections achieved through the rapid deployment of vaccines, the search for effective treatments and preventative measures against coronaviruses, including SARS-CoV-2, continues to be an active area of research [6]. Naturally occurring antiviral compounds from botanical sources offer promising yet underutilized therapeutic potential [7,8,9]. Studies have shown that natural products, such as silymarin or myricetin, interfere with the mechanisms underlying coronavirus replication [10,11] and could potentially be used to reduce the severity of symptoms in infected individuals. Panduratin A (panA) is a phytochemical found in the roots of *Boesenbergia rotunda*, a plant species in the ginger family (Zingiberaceae) that is native to Southeast Asia and commonly used in traditional medicine [12]. Previously, panA was shown to inhibit the replication of the dengue virus by targeting the viral protease, an enzyme that is essential for the virus to replicate [9]. A previous study reported that panA exhibits anti-SARS-CoV-2 activity in Vero E6 and human airway epithelial cell lines [13]. An in silico study predicted the 2′-O-methyltransferase (MTase) enzyme as a potential target of panA [14]. Additionally, molecular docking simulations of panA and its derivatives to the main protease (MPro) of SARS-CoV-2 suggested that certain panA derivatives may possess stronger inhibitory binding [15]. Furthermore, treatments using panA-containing extracts in SARS-CoV-2 infected hamsters revealed significant reductions in lung inflammation and damage [16]. However, the potential toxicity of panA to human cardiomyocytes and any inhibitory effects of panA against SARS-CoV-2 infection in cardiomyocytes specifically have yet to be explored.

Therefore, the objective of this study is to evaluate both the cardiotoxicity and antiviral efficacy of panduratin A (panA), a plant-derived compound, against SARS-CoV-2 in human cardiomyocytes. Myocytes were derived from a previously characterized human iPS cell line, MUi019-A [17], and characterized for the expression of cardiac-specific markers and host receptors for SARS-CoV-2, followed by the development of an in vitro infectivity model. A drug discovery assay was performed for panA, a bioactive phytochemical isolated from *Boesenbergia rotunda*, and its antiviral activity was demonstrated. This system provides a physiologically relevant model of viral pathogenesis in human cardiomyocytes while enabling the early discovery of potential cardiotoxicity. 

## 2. Results

### 2.1. iPSC-Derived CMs Express Cardiac Markers and Host Receptors for SARS-CoV-2

**Figure 1:**
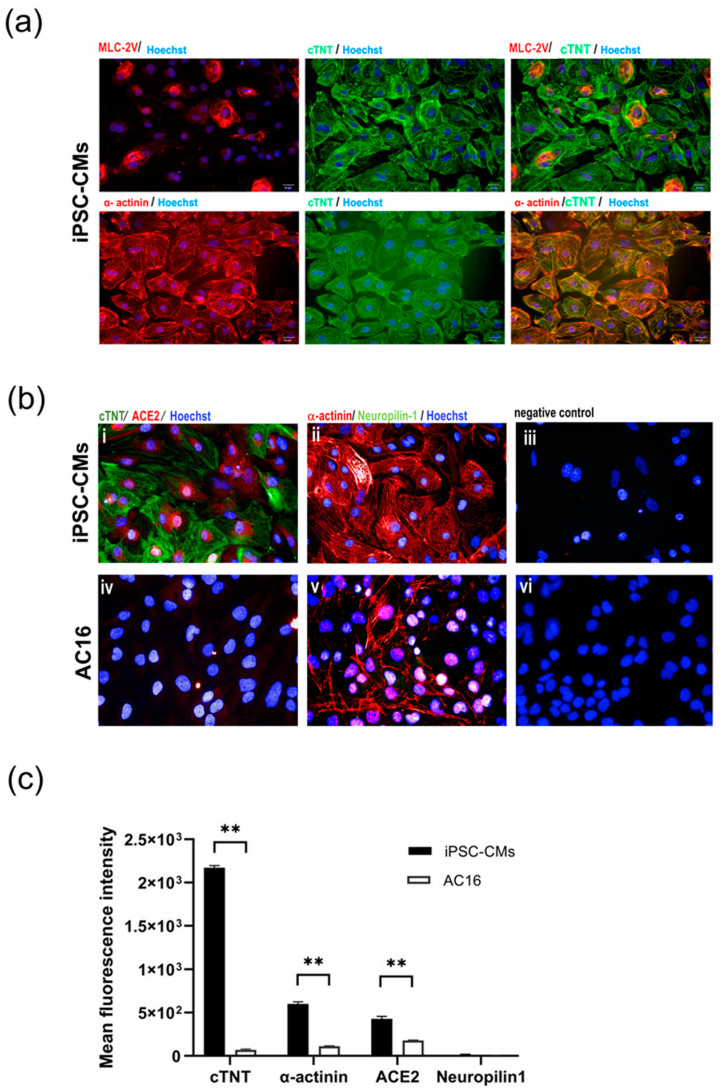
Characterization of cultured cardiac myocytes. (**a**) Expression of cardiac markers in iPSC-CMs. Differentiated myocytes (>day20) were stained for expression of cardiac trophoninT (green), α-actinin (red) and MLC2V (red), respectively. (**b**) Expression of SARS-CoV-2 host receptors in iPSC-CMs (i–iii) and AC16 myocytes (iv–vi). Differentiated myocytes (>Day20) and AC16 cells were stained for expression of SARS-CoV-2 host cell markers ACE2 (red) and neurophilin-1 (green). Nuclei were counterstained with Hoechst 33342 (blue). Cells without primary antibodies were used as controls. Scale bar 50 µm. (**c**) Mean fluorescence intensity of cardiac markers (cTNT, α-actinin) and SARS-CoV-2 host receptors (ACE2, neuropilin-1) in iPSC-CM and AC16 cells. Statistical significance was analyzed using independent Student’s *t*-tests. *p* value < 0.01 was considered significant (**).

Myocytes subjected to the directed differentiation protocol [14] were derived from the human induced pluripotent stem cell (iPSC) line MUi019-A, and the expression of cardiac markers and host receptors relevant to SARS-CoV-2 infection, namely ACE2 and neuropilin-1, were evaluated. The iPSC-derived cardiomyocytes (iPSC-CMs) exhibited robust expression of cardiac markers, including cardiac troponin T (cTNT) and α-actinin. Additionally, a marker specific to ventricular myocytes, myosin light chain-2V (MLC-2V), was detected in a small subset of the iPSC-CMs, as illustrated in Figure 1a. Notably, the iPSC-CMs also displayed the presence of ACE2, which is a crucial host receptor for SARS-CoV-2, although neuropilin-1 expression was not detected. In sharp contrast, the immortalized myocyte cell line AC16 showed α-actinin expression but did not exhibit the expression of cTNT. The AC16 cells barely expressed or entirely lacked the SARS-CoV-2 host cell markers ACE2 and neuropilin-1, suggesting their limited susceptibility to SARS-CoV-2 infection (Figure 1b). A statical analysis of the mean fluorescence intensities of cTNT and α-actinin confirmed that the iPSC-CMs had a significantly higher fluorescence intensity than the AC16 cells. Similarly, ACE2 expression was significantly higher in iPSC-CMs than the AC16 cells (*p* < 0.01), indicating a possible significant function for it in viral susceptibility. Interestingly, neuropilin-1 expression did not significantly differ between the two cell types (*p* > 0.05) (Figure 1c). 

### 2.2. iPSC-Derived CMs Are Susceptible to SARS-CoV-2 Infection In Vitro

**Figure 2:**
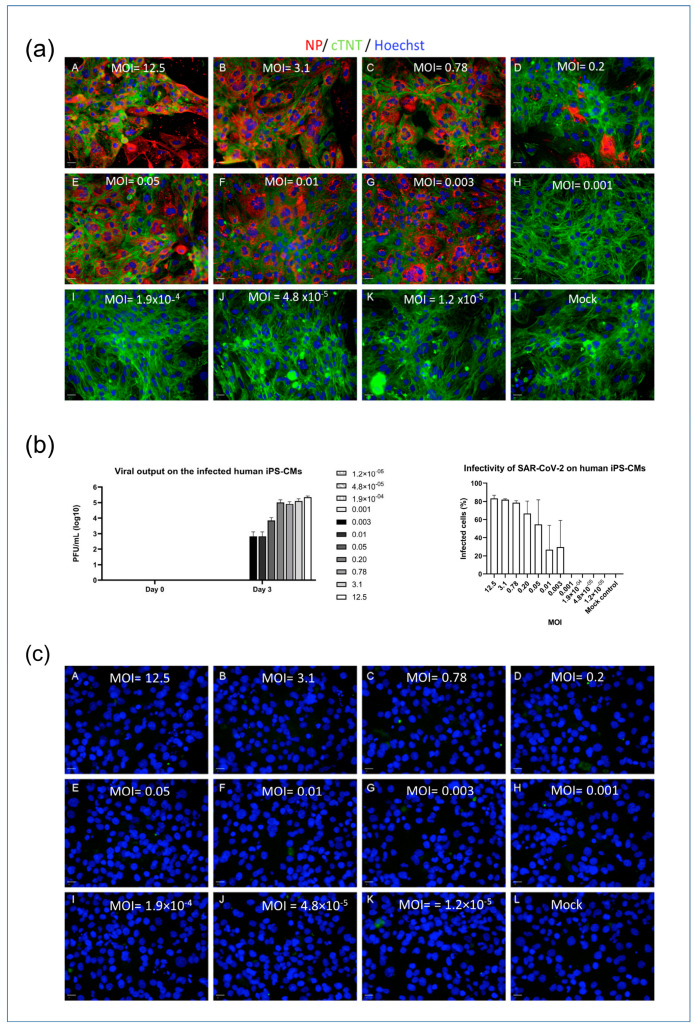
SARS-CoV-2 infectivity model of iPSC-CMs and AC16 cells. (**a**) iPSC-CMs (MOI: A = 12.5; B = 3.1; C = 0.78; D = 0.2; E = 0.05; F = 0.01; G = 0.003; H = 0.001; I = 1.9 × 10^−4^; J = 4.8 × 10^−5^; K = 1.2 × 10^−5^; L = Mock). Scale bar 50 µm. (**b**) Viral output of infected iPSC-CMs at different MOIs (left panel) and infectivity of SAR-CoV-2 on iPSCM (right panel). (**c**) AC16 cells (A = 12.5; B = 3.1; C = 0.78; D = 0.2; E = 0.05; F = 0.01; G = 0.003; H = 0.001; I = 1.9 × 10^−4^; J = 4.8 × 10^−5^; K = 1.2 × 10^−5^; L = Mock). Scale bar 50 µm. Data in figure (**b**) are represented as mean ± SEM values (n = 3 technical replicates), and values on the chart legend of figure (**b**) (left panel) are MOIs. Cells were inoculated with the SARS-CoV-2 virus, and infected cells were determined via the presence of NP (red). Microscopy images (10×) were captured 3 days post infection. A viral plaque assay was used to measure the viral output.

To investigate the infectivity of SARS-CoV-2 in different cell types, an in vitro model was employed using myocytes (iPSC-CMs and AC16 cells). The cells were seeded in 96-well plates and exposed to varying concentrations of the SARS-CoV-2 virus, indicated by MOI (multiplicity of infection) values ranging from 1.2 × 10^−5^ to 12.5. Immunofluorescence detection of the nucleocapsid protein (NP) was used to visualize viral infection within the cells, and a viral plaque assay was used to quantify the concentration of viral output at different MOIs. The iPSC-CMs displayed susceptibility to SARS-CoV-2 infection at MOI levels above 0.001, which was demonstrated by the presence of NP and viral loads detected through immunofluorescence staining and plaque assays, as depicted in Figure 2a,b. The higher the MOI level, the greater the observed viral infection within the iPSC-CMs. In contrast, the AC16 cardiomyocytes exhibited no signal of NP post infection (Figure 2c). This lack of NP detection is consistent with previous experiments that demonstrated the absence of viral receptors on the surfaces of AC16 cells. These findings suggest a differential susceptibility to SARS-CoV-2 infection between iPSC-CMs and AC16 cells, indicating that the cellular context plays a crucial role in the infectivity of the virus.

### 2.3. iPSC-Derived CMs-Based Platform Provides Accurate Screening for Cardiac Damage

**Figure 3:**
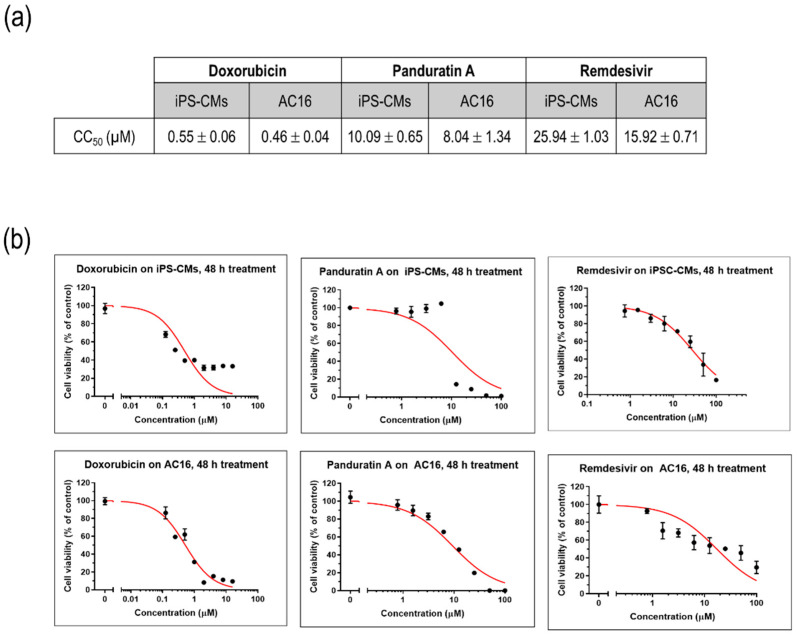
Modeling drug sensitivity and toxicity screening in cultured myocytes. (**a**) CC_50_ values of doxorubicin, remdesivir, and panA. Data are represented as mean ± SEM values (n = 3 independent experiments). (**b**) Representative figures illustrating the cytotoxic effects of doxorubicin, remdesivir, and panA at various concentrations (0–100 µM) on human induced pluripotent stem cell-derived cardiomyocytes (iPS-CMs) and AC16 cells using an MTT cell viability assay. The percentages of cell viability after 48 h of treatment relative to the non-treated control (set as 100%). Each data point (black dot) indicates as mean ± SD values (n = 3 technical replicates). The red line represents the 50% cytotoxic concentration (CC_50_).

To assess the impact of various compounds on the viability of cultured myocytes, iPSC-CMs and AC16 cells were treated with different concentrations of doxorubicin, a well-known cardiotoxic compound, as well as remdesivir, an antiviral agent commonly used in the treatment of SARS-CoV-2. A plant-derived phytochemical panduratin A (pan A), was included in the evaluation for its potential toxicity effects. After 48 h of treatment, the viability of the cells was assessed by measuring their metabolic activity using the MTT assay. The CC_50_ values, which represent the concentration of a compound that reduces the cell viability by 50%, were determined. Specifically, the CC_50_ value for doxorubicin was found to be 0.55 µM, indicating its significant toxicity towards iPSC-CMs. In comparison, the CC_50_ value for remdesivir was 25.94 µM, suggesting a relatively higher tolerance of iPSC-CMs to this antiviral agent, while panA displayed a higher toxicity with a CC_50_ value of 10.09 µM (Figure 3a,b).

### 2.4. Panduratin A Potentially Inhibits SARS-CoV-2 Infection in iPSC-Derived CMs

**Figure 4:**
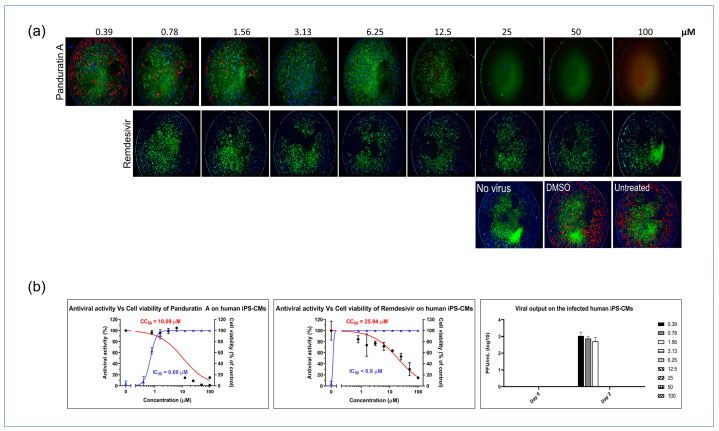
In vitro inhibition of SARS-CoV-2 in iPSC-CMs. (**a**) Myocytes were infected with SARS-CoV-2 in treatments using different concentrations of panA, remdesivir, or a dimethyl sulfoxide (DMSO) control. At 48 h post infection, cells were fixed and subjected to immunofluorescent staining by employing anti-NP (red) rabbit sera. The nuclei were stained with Hoechst dye (blue). (**b**) The left and middle panels show an evaluation of the anti-SARS-CoV-2 activity of panA. The graph shows the dose–response relationship, with the blue line indicating a 50% inhibition concentration (IC_50_) of 0.69 μM, and the red line representing the cytotoxic concentration that affects 50% of the cells (CC_50_) after 48 h of treatment, which was determined to be 10.09 μM. Each data point (black dot) refers to the mean ± SD (n = 3 technical replicates). (**b**) Right panel, illustration of viral output observed in SARS-CoV-2-infected iPS-CMs following treatment with panA at various concentrations (right symbols, µM). Error bars in the graphs represent SEM values (n = 3 technical replicates).

Next, we investigated the antiviral potential of panA in iPSC-derived cardiomyocytes infected with SARS-CoV-2. PanA demonstrated significant antiviral activity, inhibiting viral infectivity at concentrations above 0.78 µM, which is 10-fold lower than its CC_50_ value. Also, treatment with panA at concentrations higher than 0.78 µM effectively suppressed infectious virion production, as confirmed by a plaque assay analysis. These findings highlight the strong inhibitory effect of panA toward viral infection in cardiac cells, suggesting its potential as a promising therapeutic agent against SARS-CoV-2. In comparison, the antiviral agent, remdesivir, exhibited inhibitory activity against SARS-CoV-2 at 0.78 µM (Figure 4a,b).

## 3. Discussion

Induced pluripotent stem cells (iPSCs) hold great promise in the field of regenerative medicine and disease modeling due to their ability to differentiate into various cell types, including cardiomyocytes. iPSC-derived cardiomyocytes provide a unique and scalable platform for studying cardiac biology and diseases, including their susceptibility to viral infections such as SARS-CoV-2, the causative agent of COVID-19 [3]. In our study, we differentiated iPSCs into functional cardiomyocytes, which displayed the expression of important cardiac markers, such as cardiac troponin T (cTNT), α-actinin, and MLC-2V.

The expression of the host receptor necessary for SARS-CoV-2 entry into iPSC-derived cardiomyocytes is of particular interest as it allows for an investigation of viral infectivity and a study of the underlying mechanisms of cardiac involvement in COVID-19 infection [18]. In comparison, the AC16 cardiomyocyte cell line, commonly used in cardiovascular research, displayed α-actinin expression but lacked expression of cTNT and the host receptors for SARS-CoV-2 (ACE2 and neuropilin-1). This highlights the advantage of using iPSC-derived cardiomyocytes as a more physiologically relevant model for studying SARS-CoV-2 infection and its impact on cardiac cells.

Utilizing our iPSC-derived cardiomyocyte model, we sought to evaluate potential antiviral compounds for their efficacy against SARS-CoV-2. One compound of interest was panA, a natural product found in the root of *Boesenbergia rotunda*, which has been reported to possess various biological activities, including anti-inflammatory, antioxidant, and antiviral properties [9]. While remdesivir is currently approved for COVID-19 treatment, information on its safety profile remains limited, especially regarding potential drug-induced arrhythmogenic risk [19]. Our platform enabled side-by-side quantification of remdesivir’s antiviral potency and cardiac cell damage induction. Although panA’s antiviral activities were reported previously [13], its specific efficacy against SARS-CoV-2 infection in a human cardiomyocyte context has not been explored. Here, we demonstrate for the first time panA’s potent anti-SARS-CoV-2 efficacy in human iPSC-derived cardiomyocytes. We also defined a cytotoxicity threshold in these cardiomyocytes with a CC_50_ value of ~10 μM. Notably, panA elicited the sub-micromolar inhibition of wild-type SARS-CoV-2 infection in human cardiomyocytes, with antiviral IC_50_ values approximately 10-fold below its in vitro toxicity levels. This suggests that panA has the potential to be a promising candidate for further development as an antiviral therapy for COVID-19.

Antiviral properties of phytochemicals against coronaviruses are widely reported. Glycyrrhizin and lycorine, bioactive compounds derived from Chinese liquorice (*Glycyrrhiza uralensis* Fisch) and *Lycoris radiata*, respectively, have demonstrated potent inhibitory activity towards coronaviruses, supporting the potential of phytochemicals to prevent viral infections [20]. Quercetin, a flavonoid found in various plants and foods, including its derivative quercetin 3-β-galactoside, has been shown to inhibit the activity of the SARS-CoV 3CLpro protease in vitro [21]. Though panA exhibited promising antiviral properties, cytotoxicity was observed at concentrations above 10 μM, indicating further optimization of therapeutic dose ranges. This cardiotoxicity risk at higher doses remains a key limitation. Specifically, the 10-fold lower IC_50_ relative to the CC_50_ denotes a narrow window between antiviral efficacy and potential cardiomyocyte damage. Strategies to widen this gap could investigate structural analogs or modified formulations of panA to improve its selectivity and maximize antiviral potency over toxicity. Structure–activity analyses could identify pharmacological optimizations to eliminate or minimize binding to any cardiac ion channels that may disrupt electrophysiological functioning. Additional limitations of the current study involve the lack of in vivo validation and uncertainty regarding panA’s antiviral mechanism. Elucidating the molecular targets and signaling pathways involved in its inhibitory activity could suggest structural modifications to enhance its potency and safety profile. Moreover, testing panA’s antiviral effects in animal models remains imperative before clinical translation. Overall, extensive additional screening is essential to harness panA’s therapeutic benefits while preventing potential cardiotoxicity through careful dose optimization or analogue development.

The findings from our study highlight the potential of iPSC-derived cardiomyocytes for in vitro drug screening, including modeling cardiac biology, viral infection, and drug effects. Critically, while panA displayed antiviral activity at sub-micromolar levels, cytotoxicity emerged at ~10 μM. Hence, panA’s promising SARS-CoV-2 inhibition warrants careful optimization of dosing ranges to evade cardiotoxicity risks. Additionally, further efforts should focus on fully illuminating panA’s antiviral mechanism, pharmacokinetic properties, and possible toxicity mechanisms, including arrhythmogenic effects, prior to translational works. Overall, this platform enabled the recognition of panA’s antiviral potential together with insights regarding concentration-dependent toxicity—both factors requiring consideration to responsibly harness its benefits while mitigating its risks.

## 4. Materials and Methods

### 4.1. Compounds

Pure panA was isolated from a *B. rotunda* extract by the Excellent Center of Drug Discovery, Mahidol University. The identification of panduratin A and bioactivity was previously reported to inhibit viral infection in Vero cells [13]. Remdesivir and doxorubicin were purchased from Selleck chemical (Houston, TX, USA) and LC laboratories (Woburn, MA, USA), respectively.

### 4.2. iPSC Culture and CM Differentiation

Human iPS cells were maintained in E8 media on Matrigel-coated tissue culture plates (Corning). The cells were passaged routinely with EDTA, as described previously [22]. IPSC-derived cardiomyocytes were generated according to published protocols [23]. In this method, the initiation of mesoderm specification was achieved by employing 6 µM of CHIR99021 GSK3β inhibitor (LC laboratories, Woburn, MA, USA), followed by the use of 2 µM of Wnt-C59 Wnt inhibitor (Medchemexpress, Monmouth Junction, NJ, USA) to induce cardiac differentiation. A metabolic selection method involving glucose deprivation was employed to specifically isolate cardiomyocytes from the rest of the differentiated cells, as previously outlined [24]. Subsequently, the hiPSC-CMs were re-plated in a monolayer format with a density of 30,000 cells per well in hiPSC-CM culture medium (RPMI 1640 (Cytiva, Marlborough, MA, USA) supplemented with B27 (Gibco, Thermo Fisher Scientific, Waltham, MA, USA) in Matrigel-coated 96-well plates.

### 4.3. SARS-CoV-2 Infection of Cultured Myocytes

All experiments involving the infection of SARS-CoV-2 were carried out exclusively in a biosafety level 3 facility at Mahidol University. SARS-CoV-2 (SARS-CoV-2/01/human/Jan2020/Thailand) was isolated and propagated according to the previous study [13]. The viral strain was passaged once in Vero-E6 cells (ATCC, Manassas, VA, USA), and aliquots of viral stocks were prepared and stored at −80 °C. The viral titer was determined through the TCID_50_ assay using Vero-E6 cells. Vero-E6 cells were maintained in DMEM growth medium supplemented with 10% fetal bovine serum, 2 mM of L-glutamine, penicillin (100 units/mL), streptomycin (100 units/mL), and 10 mM of HEPES (Thermo Fisher, Waltham, MA, USA). These cells were incubated at 37 °C with 5% CO_2_. For the infection of myocytes, a viral inoculum was prepared using serum-free media. The culture media from each well were removed and replaced with 250 µL of the prepared viral inoculum. In the case of mock infection, 250 µL of serum-free media was added to each well. The inoculated plates were then incubated at 37 °C with 5% CO_2_ for a duration of 48–72 h before subsequent analyses.

### 4.4. Imaging and Immunofluorescence

After 48–72 h of SARS-CoV-2 infection or mock treatment, myocytes were fixed with 4% paraformaldehyde in PBS for 20 min. The fixed samples were permeabilized and blocked with a solution of PBS containing 2% bovine serum albumin and 0.3% Triton X-100 for 1 h. Primary antibodies (α-actinin, cTnT, MLC-2V (Abcam, Cambridge, MA, USA), SARS-CoV-2 nucleocapsid protein (NP) (Sino biological, Beijing, China), and neuropilin-1 (Santa cruz biotechnology, Dallas, TX, USA) were diluted in the blocking solution and added to the samples overnight at 4 °C. Following 5 rinses with PBS containing 0.3% Triton X-100, the samples were incubated with fluorescent-conjugated secondary antibodies (donkey anti-rabbit Alexa Fluor-488, goat anti-mouse Alexa Fluor-568 (Thermo Fisher, Waltham, MA, USA) diluted 1:1000 in a blocking buffer for 2 h at room temperature. Immunofluorescence images were captured using an Operetta imaging system (PerkinElmer, Shelton, CT, USA). Hoechst 33342 (Sigma-Aldrich, St. Louis, MO, USA) staining was used to count total cell numbers and determine the percentage of NP-positive cells.

For quantitative viral susceptibility assays, virus- or mock-infected cells were fixed and subjected to co-staining with specific antibodies against cardiac troponin T (cTnT) and the SARS-CoV-2 nucleoprotein (NP). Subsequently, appropriate secondary antibodies and Hoechst nuclear staining dye were applied for visualization. The stained cells were then imaged using a high-content imaging system (Operetta, PerkinElmer). To quantify the results, the percentages of infected cells (NP positive) and myocytes (cTnT positive) in each well were automatically captured and analyzed using Harmony software version 4.1 (PerkinElmer, Shelton, CT, USA). 

### 4.5. Cytotoxicity Assays

In vitro cardiotoxicity assays were conducted to assess cell viability using the MTT (3-(4,5-dimethylthiazol-2-yl)-2,5-diphenyltetrazolium bromide) assay, as previously described [25]. At 48 h after drug treatment, the cell culture medium was removed, and an MTT solution was added and incubated at 37 °C for 3 h. After incubation, the MTT solution was removed, and the formazan crystals formed within viable cells were dissolved using dimethyl sulfoxide (DMSO, PanReac Applichem, Chicago, IL, USA). The absorbance at 570 nm and 690 nm was measured using a microplate reader (Versamx, Molecular Devices, Chicago, IL, USA).

### 4.6. Micro-Plaque Assay

To assess the replication capacity of SARS-CoV-2 in human cardiomyocytes, a micro-plaque assay was performed. In this assay, Vero cells were inoculated with a serial dilution of the 42 or 72 h post-infection supernatant and incubated for viral adsorption at 37 °C for 2 h. The cells were then treated with MEM media containing 5% fetal bovine serum (FBS) and 1% agarose, followed by further incubation at 37 °C with 5% CO_2_. After 24 h post infection, the cells were stained with a rabbit anti-nucleoprotein (NP) antibody and a goat anti-rabbit horseradish peroxidase (HRP) antibody (Dako). The SureBlue substrate (Merck) was added and allowed to develop for 10 min. The positive colonies formed were counted as plaque-forming units per milliliter (PFUs/mL), a measure of viral output.

### 4.7. Data and Statistical Analyses

Dose–response curves for each compound were analyzed, and the 50% cytotoxic concentration (CC_50_) and 50% inhibitory concentration (IC_50_) were calculated using GraphPad Prism 10 (GraphPad Software), using the non-linear regression equation with the inhibitor concentration vs. a normalized response model. Independent Student’s *t*-tests were performed to compare the significant difference between iPS-CMs and AC 16 cells, and *p* < 0.01 was considered statistically significant (**). Results of the data analysis are presented as mean ± SD or mean ± SEM values.

## Data Availability

All data analyzed in this study are included in this article.
